# Predictive modeling of burnout based on organizational culture perceptions among health systems employees: a comparative study using correlation, decision tree, and Bayesian analyses

**DOI:** 10.1038/s41598-024-56771-2

**Published:** 2024-03-13

**Authors:** Teray Johnson, Sameh Shamroukh

**Affiliations:** 1 Data Sciences, Harrisburg University of Science and Technology, 326 Market Street, Harrisburg, PA 17101 USA; 2 Data Sciences, Harrisburg University of Science and Technology, Harrisburg, PA USA

**Keywords:** Burnout, Health systems, Administrative employees, Organizational culture, COVID-19 pandemic, Health care, Occupational health

## Abstract

Burnout is a significant concern, particularly within the healthcare field, affecting both nurses and physicians. It is a common issue in health systems, which encompass a range of healthcare facilities, such as hospitals, physician practices, ambulatory sites, and administrative offices like finance. Despite this, there has not been an extensive exploration of burnout in employees working directly with patients versus those in non-patient-facing roles within these health systems. It is important to note that organizational culture plays a crucial role in influencing various aspects of employees' work-life balance and their experiences of burnout. This study adopts a cross-sectional design, involving the distribution of a 57-question Likert scale survey to employees in health systems. These employees serve in various roles, both patient-facing and non-patient-facing, within jointly owned healthcare organizations, which encompass hospitals, ambulatory sites, and administrative offices. The survey was disseminated through trade organizations and employees at the managerial level and above within these health systems. Data was collected between October 2022 and January 2023, resulting in a total of 67 responses. The study employs correlation analysis to explore the connection between organizational culture and burnout. Furthermore, a decision tree model is constructed to predict burnout scores based on survey responses, specifically the question regarding the perceived positivity of the organizational culture. The decision tree models indicate that perceiving organizational culture as positive, safety-oriented, and supportive predicts various outcomes for individuals, including job retention, positive experiences with patients, increased callousness, and stimulation while working with colleagues. Bayesian analysis, considering the small sample size, reinforces these findings and provides a different perspective, incorporating prior knowledge and credible intervals. An association test suggests a strong link between a positive organizational culture and burnout symptoms, while another test supports a connection with engagement signs. Similar to nurses and physicians, administrative health systems’ personnel are susceptible to burnout. Organizational culture can affect burnout. Therefore, health systems’ leaders should cultivate an organizational culture that protects against burnout.

## Introduction

Organizational culture (OC) is a factor in every workplace. Schein defines OC as including tangible artifacts, the organization’s values and beliefs, and underlying assumptions that lie and operate unconsciously within an organization’s employees^1,2^. These assumptions influence employees’ behavior. OC can originate and be perpetuated by an organization’s leaders^3^. Due to the potential of OC to influence behavior, leaders should study how to cultivate and maintain a positive OC.

Aside from its impact on behavior, organizational culture (OC) can have implications for burnout rates. Burnout encompasses three dimensions: exhaustion, characterized by feelings of being drained, depleted, and lacking energy; depersonalization, where employees exhibit negative attitudes towards clients, become irritable, lose their sense of idealism, and withdraw from interpersonal interactions; and reduced personal efficacy, signifying performance challenges and an inability to cope with problems^4^. Despite previous research highlighting the connection between OC and burnout, there is a dearth of studies that address both clinical and non-clinical employees within health systems. These health systems are collaborative healthcare organizations encompassing hospitals, physician practices, ambulatory sites, and administrative offices (e.g., finance)^5–10^. Existing research has predominantly focused on nurses and physicians when examining perceptions of OC and burnout^9,11,12^. Furthermore, there is a notable absence of a burnout prediction model that utilizes OC factors for both patient-facing and non-patient-facing health systems’ employees. Consequently, this study aims to identify the OC factors that influence burnout and investigate the relationship between OC and burnout among employees in health systems. To contribute to the existing literature, this study creates a decision tree model and correlation matrix that explores various OC aspects and burnout symptoms among both patient-facing and non-patient-facing health systems’ employees.

### Literature review

OC is composed of several factors. One factor is perceived organizational support, which includes support from leaders and transformational leaders who listen to and address employees’ concerns^13^. Other factors include teamwork, peer relationships, the ability to develop in one’s role, involving employees in decision-making, blamelessness when employees make mistakes, and how employees treat customers^14–18^. Among operating room nurses, for example, teamwork is imperative to cultivating respect among co-workers and is one indicator of a positive OC^16,19^. An open and honest environment in which co-workers are friendly and respectful toward one another is indicative of a positive OC^20^. The articles mentioned above study aspects of OC with primary focus on nurses, physicians, medical students, and other clinical staff. Therefore, more research is needed to show perceptions of OC among non-patient-facing employees in health systems.

#### The relationship between organizational culture and burnout

Numerous studies have found that a negative organizational culture can contribute to burnout. For example, a study by Maslach and Leiter found that employees who perceived their workplace culture as unsupportive were more likely to experience burnout^21^. Similarly, a study by Mäkikangas and Kinnunen found that employees who experienced high levels of organizational conflict and role ambiguity were more likely to experience burnout^22^.

Conversely, a positive organizational culture can protect against burnout. A study by Halbesleben and Buckley found that employees who perceived their workplace culture as supportive and empowering were less likely to experience burnout. Another study by Bakker, Schaufeli, Leiter, and Taris found that a positive organizational culture characterized by high levels of autonomy and social support was associated with lower levels of burnout. A third study by Malik et al. concluded that a favorable working environment is necessary to reduce the incidence of job burnout in every industry, especially the higher education sector^23^. Furthermore, O’Connor et al. found that work-related factors such as workload and work relationships are determinants of burnout^24^. Role clarity, professional autonomy, feeling fairly treated, and access to supervision protect against burnout^24^.

Among operating room nurses, OC can moderate burnout^25^. However, the authors study only nurses and a work-oriented OC, not other types of OC. A patient safety culture in which employees are not blamed for mistakes and feel able to report mistakes without fear of retaliation is negatively related to burnout^20,26^. Additionally, a lack of support from supervisors, which is one aspect of OC, can increase burnout rates^13,27^. These studies show that each of the factors as mentioned above of OC can contribute to burnout among clinical staff in health systems. However, the same might not hold true for non-patient-facing health systems’ employees. More research is needed to show whether having a positive, data-driven, supportive, and safety-oriented OC is correlated with and predicts burnout symptoms among non-patient-facing health systems’ employees.

#### Measuring organizational culture and burnout

Several authors measure OC and burnout in separate scales; however, none use a combined reliable and validated scale. Therefore, this study adapts a validated and reliable scale by Kovner et al. (2007) to measure both OC and burnout in the same instrument^28^. Furthermore, most studies about OC and burnout in health systems focus on nurses and physicians instead of administrative employees^19,29–35^. This study includes administrative, non-patient-facing employees’ perceptions of OC and experiences with burnout.

#### Burnout prediction models

In a study conducted by Kim et al., turnover rates, a consequence of burnout, were predicted through the utilization of a decision tree, random forest, and logistic regression model^36^. This study focused on nurses in Korea, using secondary data, and identified salary as the primary predictor of turnover. Notably, this prediction model did not incorporate OC variables and omitted other job roles, such as hospital administrators, from consideration.

Another study in Medan, Indonesia, found that a supportive OC had only a minimal impact on nurses' burnout rates^37^. The investigation solely explored the presence of a supportive OC, without delving into other OC factors, including safety culture. Furthermore, the study exclusively featured nurses within its sample.

Whitehead et al. employed a structural equation model (SEM) to explore the correlation between OC and hospital employee wellbeing^6^. The assessment covered multiple facets of OC, encompassing areas like creativity, innovation, and attention to employees within the organization. Despite the focus on how OC predicts wellbeing, including aspects like spiritual health and stress management, the study did not explicitly address burnout. Additionally, it did not specify the roles of the surveyed personnel, potentially leading to a bias toward patient-facing personnel, such as nurses.

Yulita & Abdullah conducted a study involving teachers in Malaysia, with the aim of evaluating whether an organization fostering a psychologically safe climate (PSC) through inclusive decision-making, effective communication, management support, and prioritization of psychological health could predict work recovery rates^5^. "Recovery" in this context referred to the ability to relax during non-work hours, and it was inversely related to emotional exhaustion, a symptom of burnout^38,39^. The study employed a hierarchical linear model to assess whether a PSC moderates emotional exhaustion, yet it did not explore whether a PSC moderates the depersonalization and personal efficacy aspects of burnout.

Unlike previous studies that do not include non-patient-facing roles in their prediction models, such as project managers, this study uses decision tree models to assess how organizational culture (OC) can predict burnout rates among patient-facing and non-patient-facing health systems’ employees. Furthermore, unlike previous studies, this study includes several OC factors, such as safety culture and employee involvement in decision-making. This study has several practical implications and will allow health systems’ leaders to better assess which aspects of OC to improve to alleviate burnout.

Based on the previously cited literature about perceptions of OC and burnout and the lack of studies including administrative health systems’ employees, the research questions are as follows:

1. Are factors of OC related to one another among health systems’ employees?
2. Are burnout symptoms related to one another among health systems’ employees?
3. How do factors of OC influence burnout among health systems’ employees?
4. Can a belief in a positive OC predict responses to burnout?

Since the literature above states that a relationship exists between OC and burnout, the dependent variables (DVs) is the OC question responses to C30. The independent variables (IVs) are the burnout question responses of B1 through B18.

The objective function is as follows:

$$ f = {\text{ Max }}\left( {{\text{C9}},{\text{ C1}}0,{\text{ C19}},{\text{ C21}},{\text{ C22}},{\text{ C3}}0} \right) \, + {\text{ B1 }} + {\text{ B2}} \ldots {\text{B18}} $$

**Figure 1:**
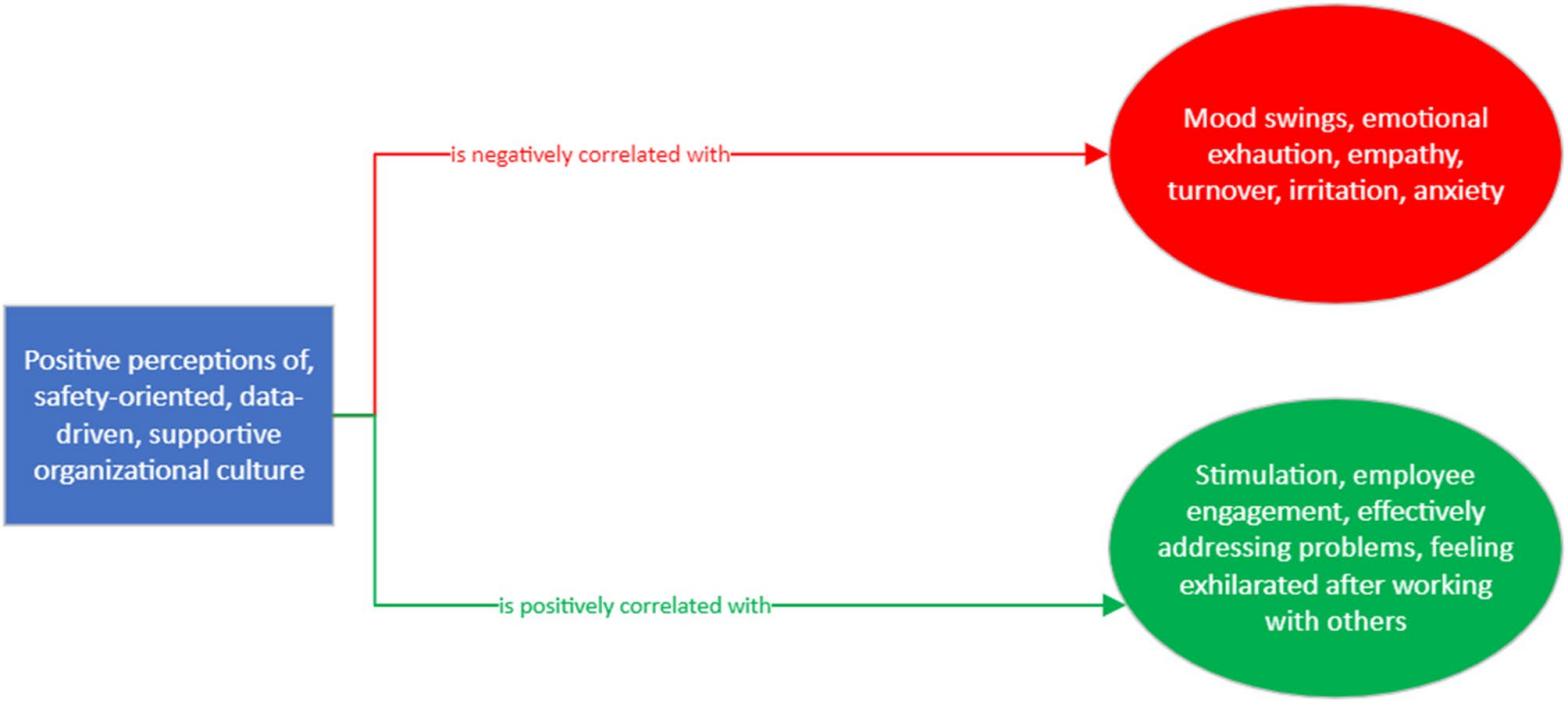
Theoretical map of the relationship between OC and burnout.

To answer each research question, the following hypotheses are studied and shown in Fig. 1:

##### H1

High scores in questions C9, C10, C19, C21, C22, and C30 are negatively correlated with burnout scores from questions B1 (feeling emotionally drained after work), B5 (job interference with home-life), B6 (job interference with family time), B7 (irritation), B8 (anxiety), B9 (mood swings), B10 (feeling on-edge), B11 (fatigue), B12 (feeling at wits’ end), B15 (treating others as impersonal objects), and B17 (feeling callous toward others).

##### H2

Low scores in question C9, C10, C19, C21, C22, and C30 are positively correlated with burnout scores from questions B2 (understanding patients’ feelings), B3 (understanding visitors’ feelings), B4 (taking one’s current job again), B13 (feeling stimulated when working with colleagues), B14 (effectively addressing problems), B16 (feeling relaxed when addressing emotional issues), and B18 (feeling exhilarated after working with or talking to patients).

## Methods

### Setting, measurement, and study design

This cross-sectional study received approval from the Harrisburg University of Science and Technology Institutional Review Board (IRB) under the reference number 20221026. Before commencing the survey, the required sample size was determined to be 385. This estimation was based on an assumed population of 2,000,000 health system workers, with the objective of generalizing the findings and ensuring adequate statistical power within a 95% confidence interval^40^. Data collection was accomplished by employing a 57-question Likert scale survey known for its validation and reliability, as developed by Kovner et al. to assess various aspects of OC and burnout^28,41^. Detailed information regarding the instrument's validation and reliability can be found in the studies conducted by Kovner et al.^28,41^. The burnout construct, which comprises 18 questions, was evaluated using a 1-to-5 point Likert scale. Within this scale, a rating of 1 signified strong disagreement with the statement, while a rating of 5 indicated strong agreement. The burnout construct further encompassed depersonalization (4 items), emotional exhaustion (7 items), and personal accomplishment (7 items).

The organizational culture construct featured 31 items distributed across various factors, including supervisor support (6 items), work-group cohesion (6 items), work attitudes (12 items), and safety (7 items). The remaining 6 items pertained to demographic information. Principal components analysis was employed to establish each construct and subscale^28^. Modifications to the scale incorporated demographic inquiries, such as the participants' department of employment and job role nomenclature.

To disseminate the survey, it was published on JotForm.com, and a link was shared with trade organizations and influential individuals within health systems. This distribution strategy was employed to minimize potential biases.

The survey implemented an informed consent process at the survey's outset, providing participants the freedom to exit at any point without facing penalties or retention of their previously submitted responses. Data collection transpired over a period from February 2022 to October 2022.

### Participants

All employees who worked for a health system (i.e., jointly owned organizations with at least one hospital and physician practice) were eligible to participate. Employees included patient-facing, non-patient-facing, management, and non-management employees to study the differences between perceptions of OC and burnout rates among each group. Eligible employees were invited to participate via trade organizations, such as hospital associations and accountable care organizations, and by contacting people of influence, such as human resources managers within health systems, who then distributed the survey to health systems’ employees. A total of 104 health systems, trade organizations, and manager-level health systems’ employees were contacted for distribution of the survey via e-mails, phone calls, and social media (e.g., LinkedIn). Health systems contacted had sizes ranging from one hospital and outpatient center to over 10 hospitals and outpatient centers.

### Analysis

All data were analyzed using R, a statistical analysis software. Since all survey questions were mandatory to answer, there were no missing data to address. The data were split into OC and burnout responses. A correlation analysis was conducted, and a decision tree model was created for the OC and burnout questions. Due to the small sample size, a Bayesian analysis was conducted^42–44^.

### Ethics approval and consent to participate

This research was approved by the Harrisburg University of Science and Technology’s Institutional Review Board (IRB# 20221026). Participants provided informed consent at the beginning of the survey. They also provided verbal and written informed consent prior to the interviews. All methods were carried out in accordance with the Declaration of Helsinki.

## Results

The survey yielded a total of 67 responses. Nevertheless, four responses originating from board members, who do not have daily involvement in the health system, were excluded from the analysis. Factor analysis was performed to assess reliability and validity, resulting in Cronbach alpha scores of 0.97 for organizational culture (OC) and 0.84 for burnout. Moreover, the OC questions exhibited a high intercorrelation of 0.89, while the burnout questions demonstrated strong correlations at 0.97. These findings, supported by the Cronbach alpha and correlation analyses of each factor, underscore the robust reliability and validity of the survey instrument^45^. Among the 67 responses, a total of 63 participants were identified as employees within health systems, rendering their responses eligible and subsequently included in the analytical process. The breakdown of participant characteristics, as presented in Tables 1, 2, and 3, reveals that a majority of participants were affiliated with health systems with over 500 beds. Furthermore, most participants held administrative roles, falling under the categories of middle- or senior-level management, and primarily operated within administrative departments, such as nursing administration. As for geographical distribution, the data illustrated that the majority of participants were employed by non-profit health systems located in urban areas.

**Table 1:** Departments.

Department	Number of respondents
Administration	20
Community health	3
Diagnostic imaging/radiology	1
ED	4
Inpatient psychiatry	1
Inpatient surgery	2
Lab services	1
Med/surg	2
OR/PACU	1
Pharmacy	1
Quality and patient safety	4
Step down/transitional	1
Analytics	4
Case management/social work	1
Finance	1
Graduate medical education	1
Information technology	2
Marketing/business Development	2
Operations	1
Strategy	5
Patient experience	1
Radiation Oncology	2
Refused to answer	3

**Table 2:** Position.

Position title	Number of respondents
Administrator/assistant administrator/supervisor	17
Consultant	4
Case manager	1
Clerical	1
IT support	2
Medical assistant	12
Medical doctor (MD)	10
Patient coordinator/access representative	3
Chaplain	1
Physician chief/chair	1
Project manager	1
Registered nurse	7
Residency coordinator	1
Other	2

**Table 3:** Hospital size, location, and type.

	Number of respondents
Bed size	
6–24 beds	3
25–49 beds	0
50–99 beds	3
100–199 beds	7
200–299 beds	3
300–399 beds	5
400–499 beds	8
500 beds or more	34
Location	
Rural	22
Urban	41
Type	
Federal	2
For-profit	16
Non-profit	45

**Figure 2:**
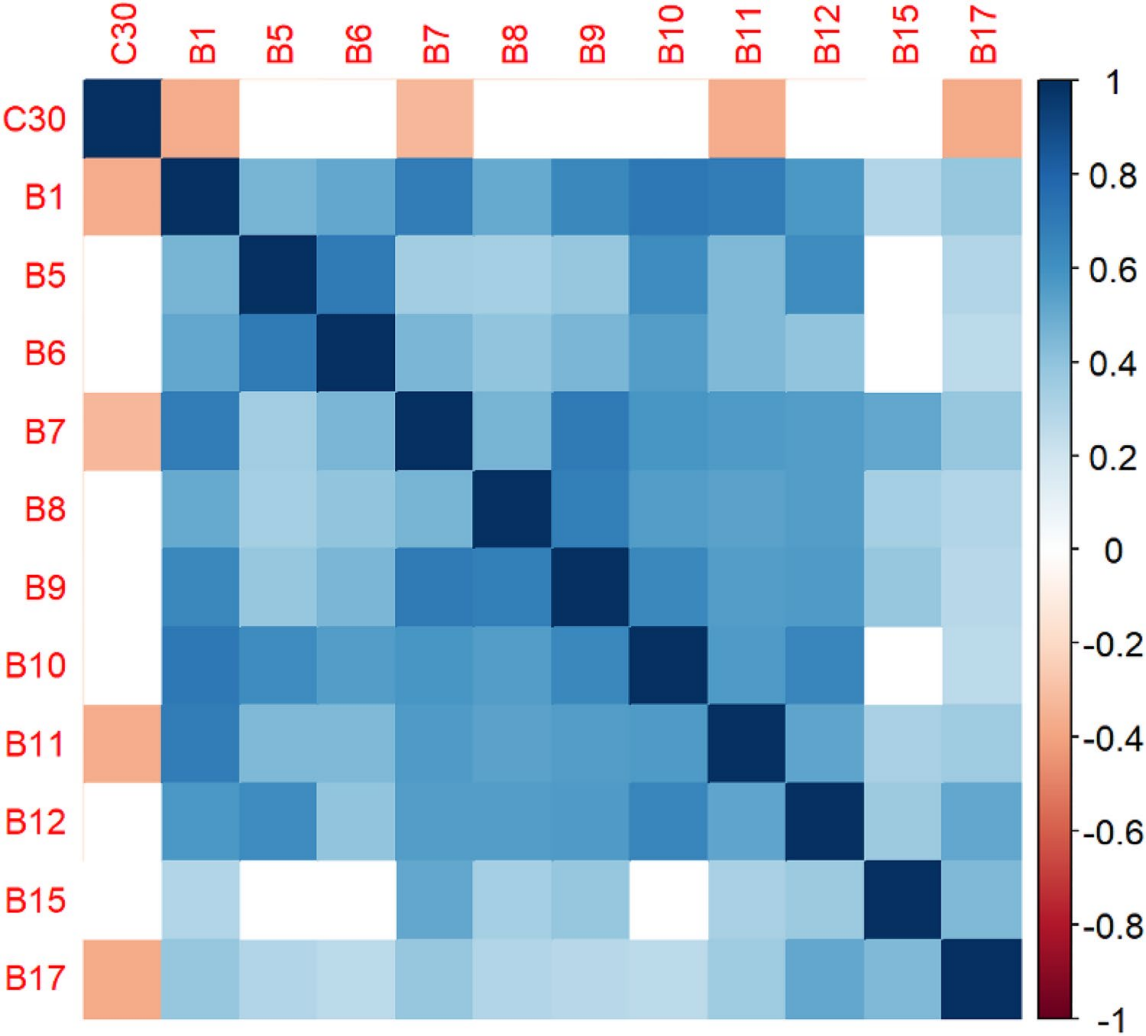
Correlation matrix between C30 and burnout items.

**Figure 3:**
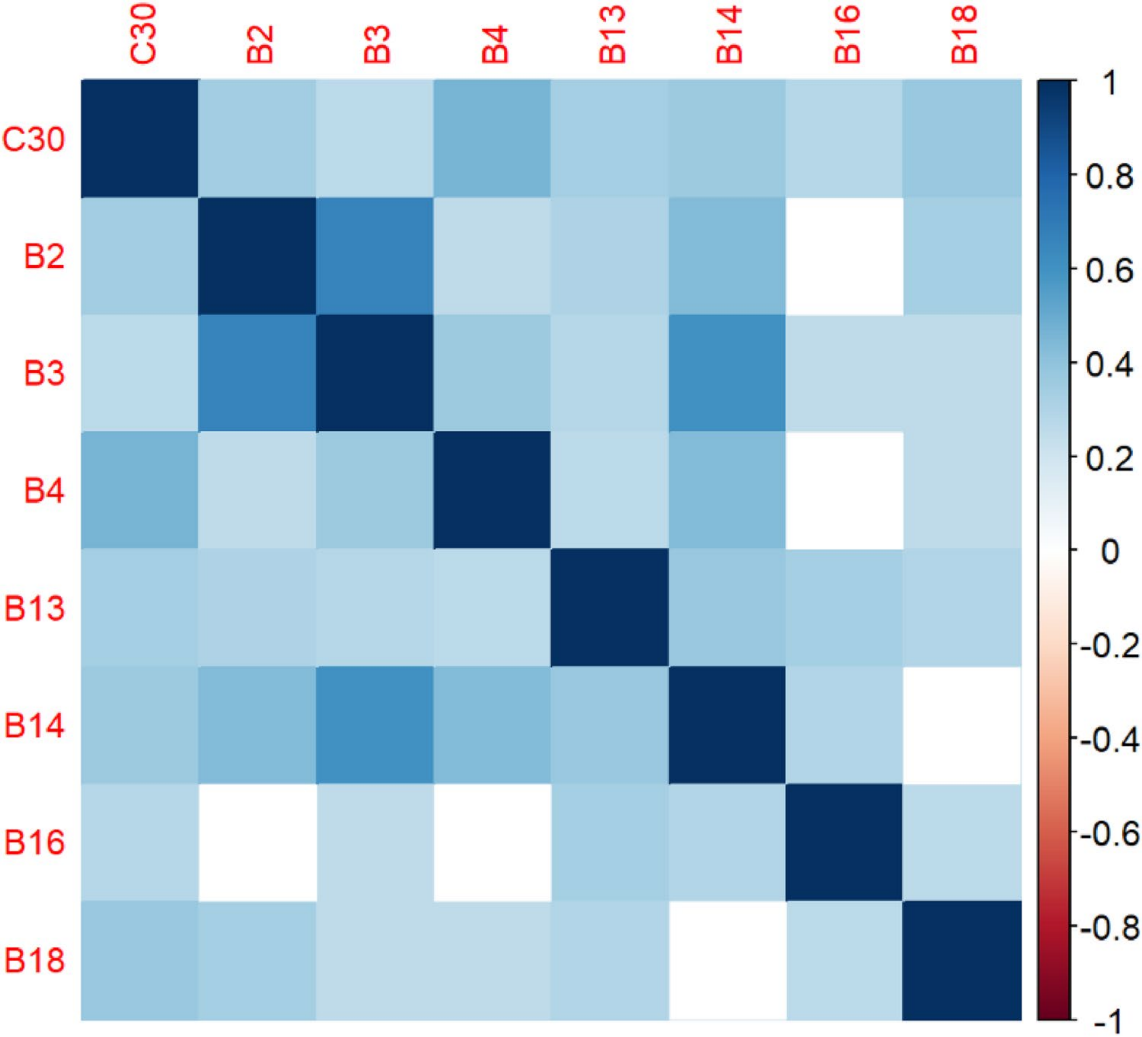
Correlation matrix between C30 and remaining burnout items.

**Figure 4:**
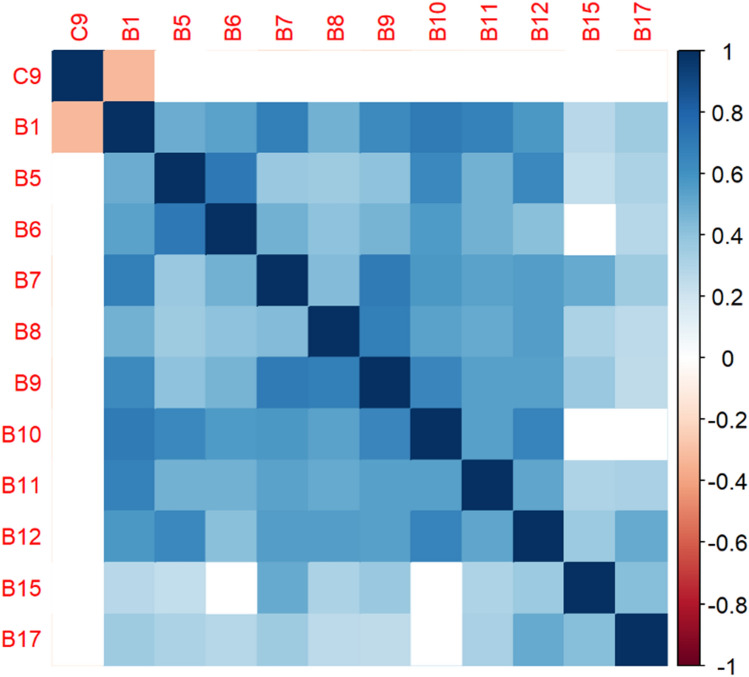
Correlation matrix between C9 and burnout items.

**Figure 5:**
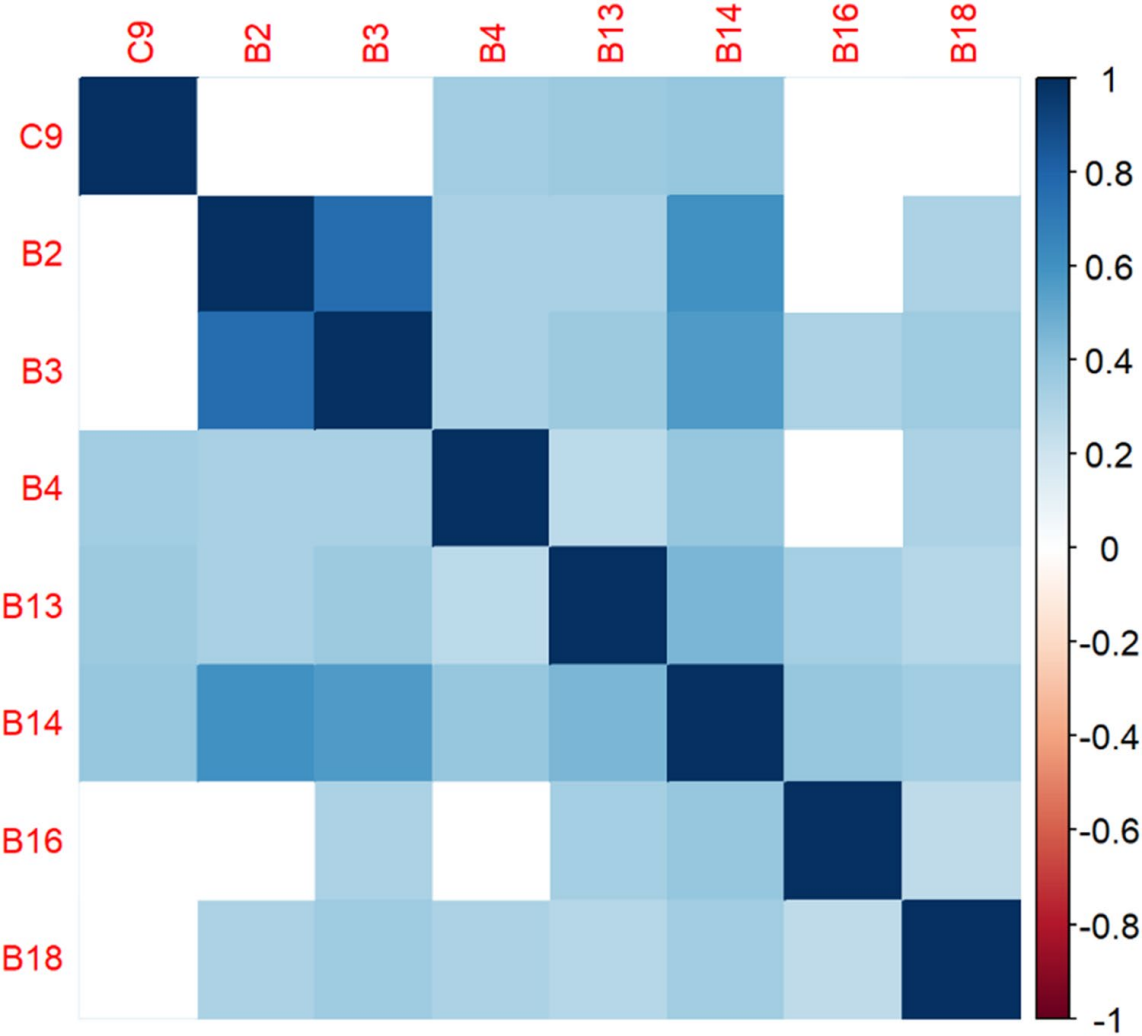
Correlation matrix between C9 and remaining burnout items.

**Figure 6:**
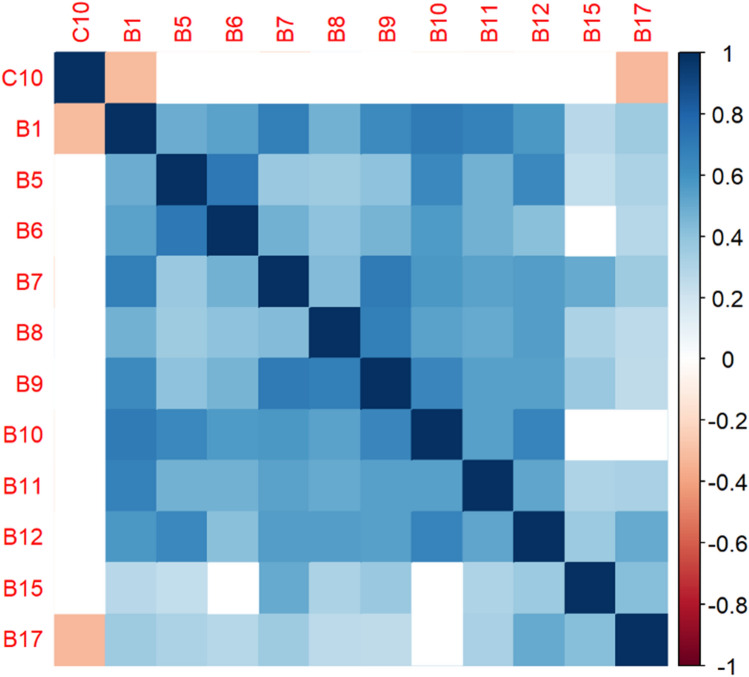
Correlation matrix between C10 and burnout items.

**Figure 7:**
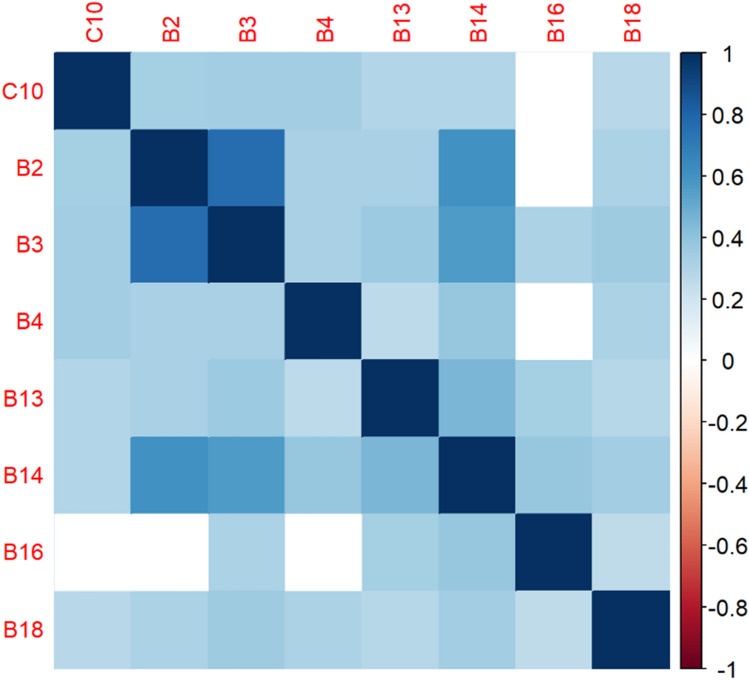
Correlation matrix between C10 and remaining burnout items.

**Figure 8:**
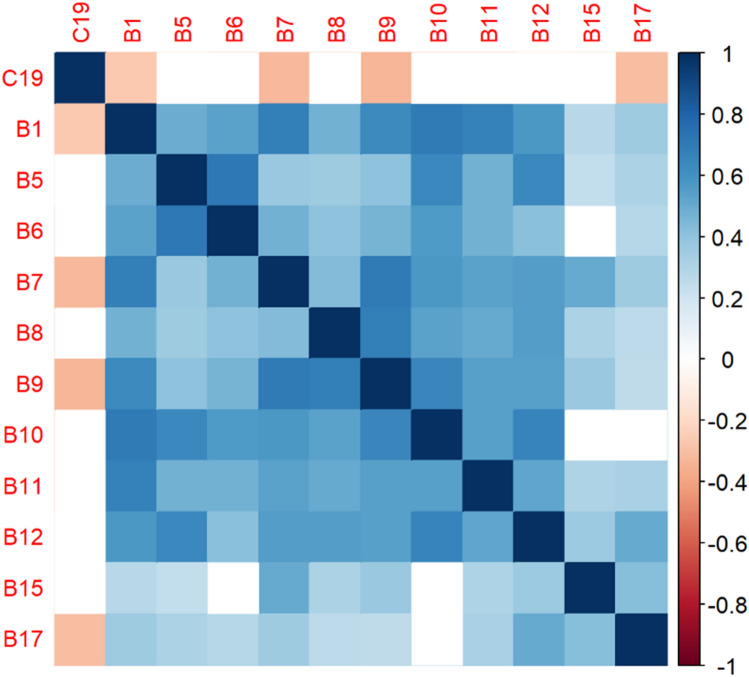
Correlation matrix between C19 and burnout items.

**Figure 9:**
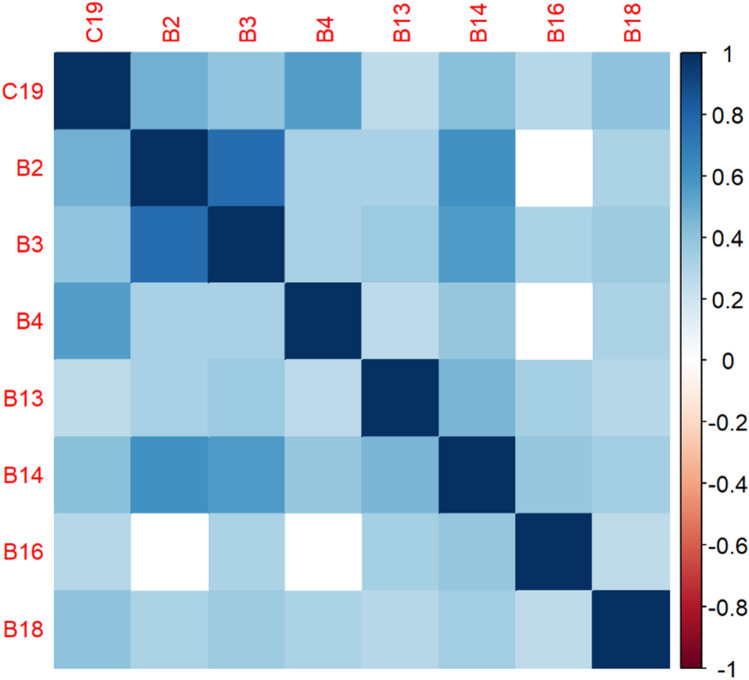
Correlation matrix between C19 and remaining burnout items.

**Figure 10:**
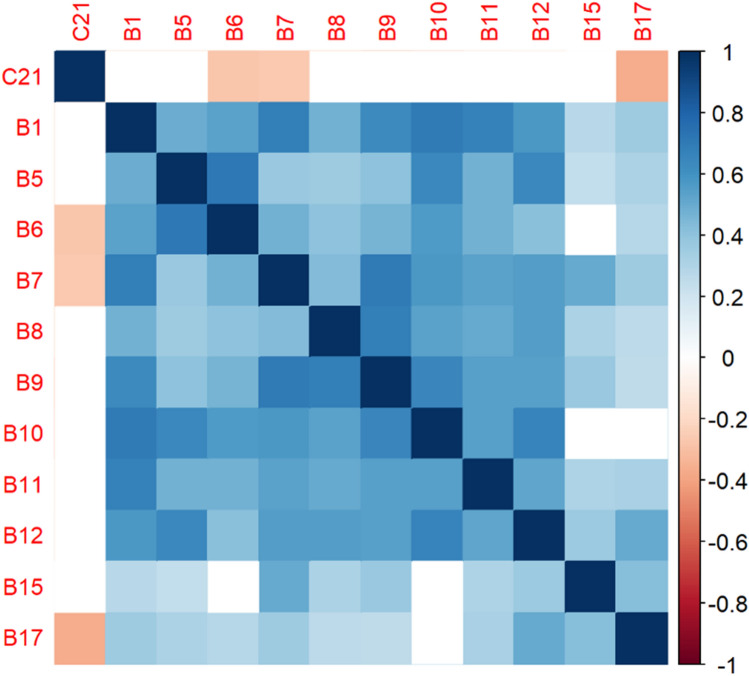
Correlation matrix between C21 and burnout items.

**Figure 11:**
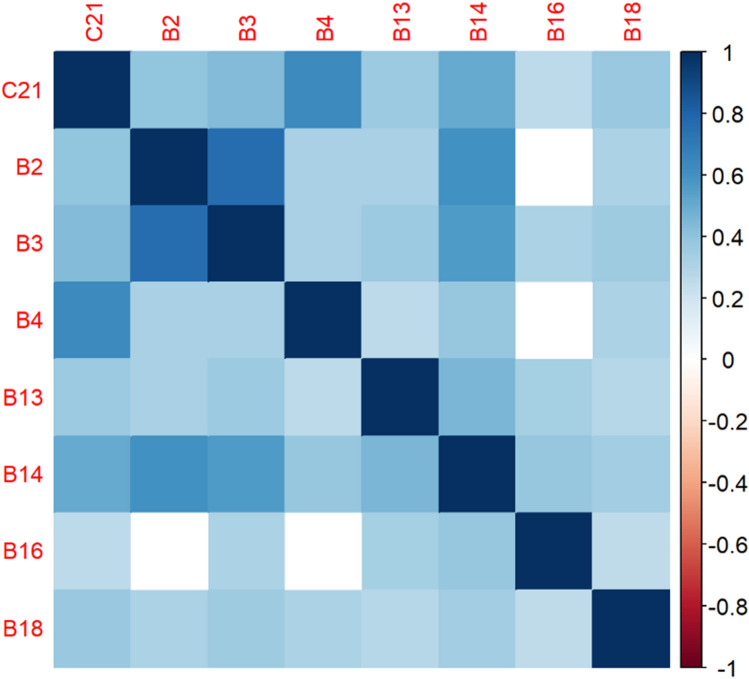
Correlation matrix between C21 and remaining burnout items.

**Figure 12:**
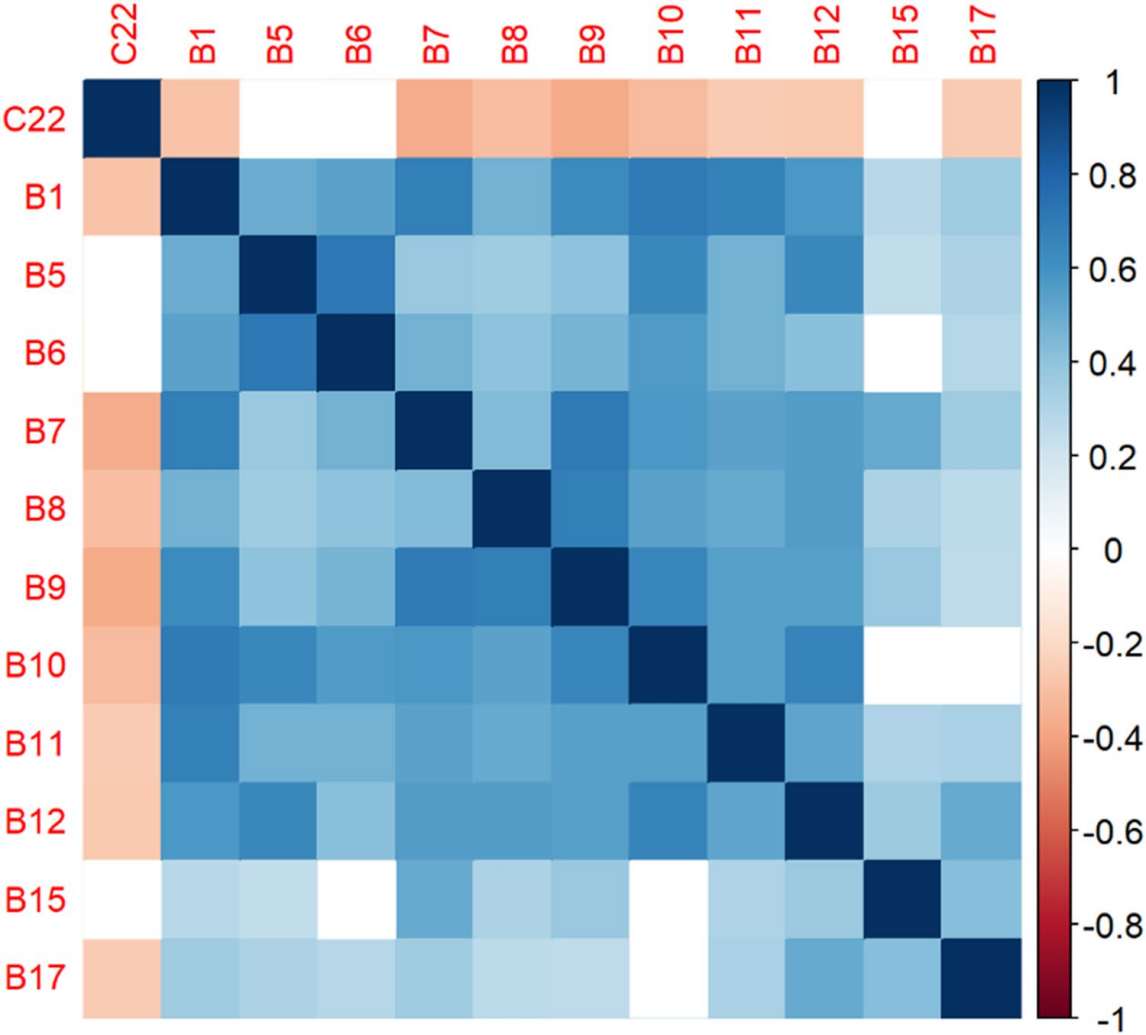
Correlation matrix between C22 and burnout items.

**Figure 13:**
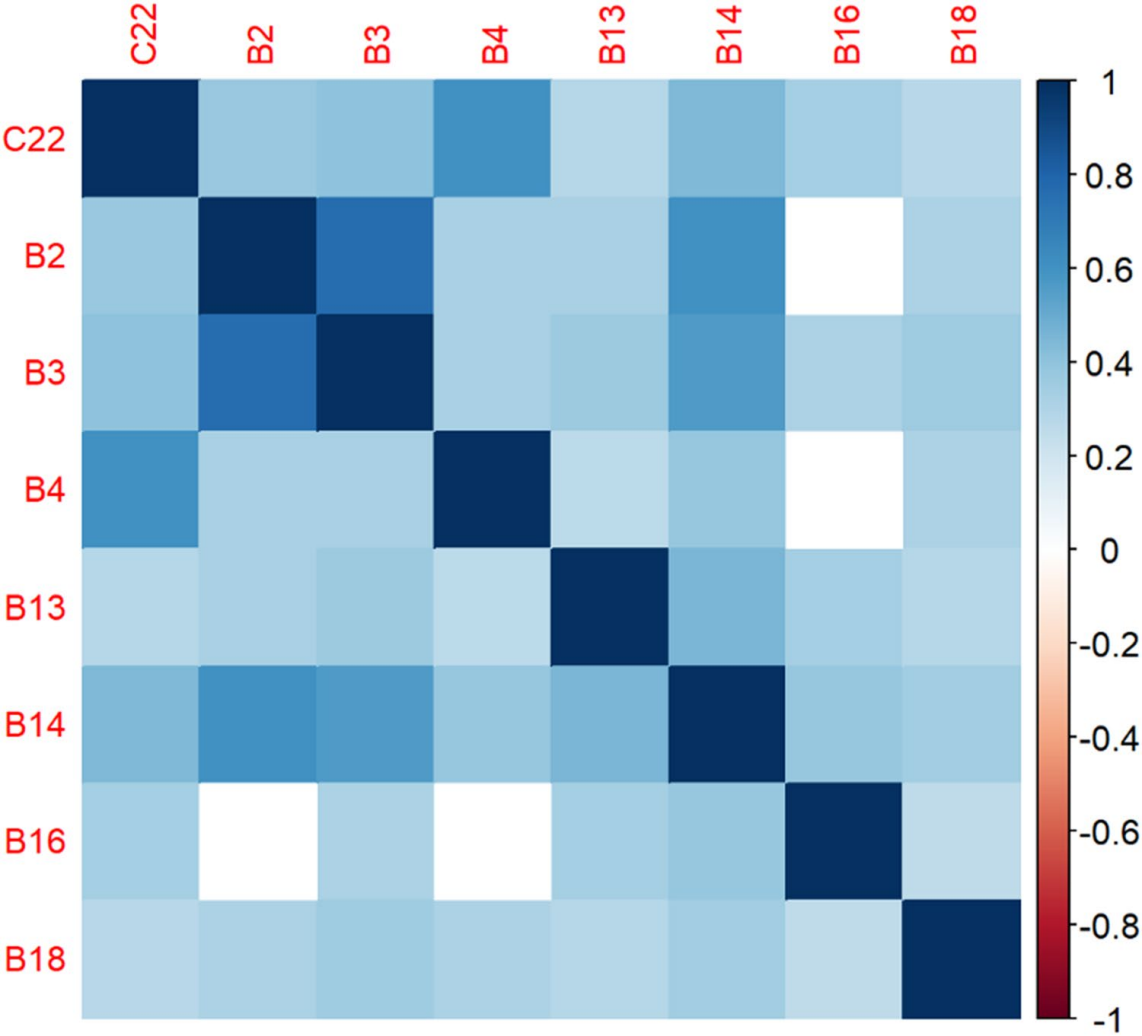
Correlation matrix between C22 and remaining burnout items.

The correlation matrices in Figs. 2, 3, 4, 5, 6, 7, 8, 9, 10, 11, 12 and 13 were created to show correlations between survey items. The matrices show statistically significant positive correlations between the belief that one’s OC is positive and taking one’s current job again, understanding patients’ and visitors’ feelings, feeling stimulated when working with colleagues, effectively addressing patients’ and co-workers’ problems, and feeling exhilarated after working with or talking to patients. The matrix shows statistically significant negative correlations between the belief that one’s OC is positive and feeling emotionally drained after work, irritation, fatigue when waking in the morning, and feeling more callous toward others since beginning one’s current job.

Employee involvement in decision-making is positively correlated with taking one’s current job again, feeling stimulated when working with colleagues, and effectively addressing patients’ and co-worker’s problems. It is negatively correlated with feeling emotionally drained after work. Data-driven decision-making is positively correlated with understanding patients’ and visitors’ feelings, taking one’s current job again, feeling stimulated when working with colleagues, effectively addressing patients’ and co-workers’ problems, and feeling exhilarated after talking or working with patients. It is negatively correlated with becoming more callous toward others since starting one’s current job and feeling emotionally drained after work. Not sacrificing patient safety to get more work done is positively correlated with understanding patients’ and visitors’ feelings, retaking one’s current job, feeling stimulated when working with colleagues, effectively addressing patients’ and co-workers’ problems, being relaxed with addressing emotional problems, and feeling exhilarated after working with or talking to patients. It is negatively correlated with being easily irritated, mood swings, becoming more callous toward others, and feeling emotionally drained after work. Having needed supplies and equipment is positively correlated with understanding patients’ and visitors’ feelings, retaking one’s current job, feeling stimulated when working with colleagues, effectively addressing patients’ and co-workers’ problems, being relaxed with addressing emotional problems, and feeling exhilarated after working with or talking to patients. It is negatively correlated with being easily irritated, becoming more callous toward others, and one’s job preventing them from spending time with family.

### Figure key

**OC Subdomain—Work-group Cohesion**

C9—Members of my work unit/department are involved in decisions that directly affect their work.

C10—Decisions are made based on research, data, and technical criteria, as opposed to political concerns.

C30—Overall, the culture of the hospital is positive.

**OC Subdomain—Safety**

C19—Patient and employee safety are never sacrificed to get more work done.

C21—I have the supplies and equipment needed to perform my job effectively and safely.

C22—As an employee, I feel comfortable reporting potential or actual patient and employee safety problems.

**Burnout Subdomain—Emotional Exhaustion**

B1—When I go home after work, I feel emotionally drained.

B7—I often get irritated by little annoyances.

B8—I suffer from anxiety.

B9—My mood often goes up and down.

B10—There are days when I'm "on edge" all the time.

B11—I feel fatigued when I get up in the morning.

B12—I feel like I'm at my wits' end.

**Burnout Subdomain—Depersonalization**

B2—I understand my patients' feelings.

B3—I understand visitors' feelings.

B15—I treat some of my patients and/or co-workers like they're impersonal objects.

B17—I've become more callous toward people since starting my current job.

**Burnout Subdomain—Personal Accomplishment**

B4—Knowing what I know now, I would take my current job all over again.

B5—My job interferes with my home-life.

B6—My job keeps me from spending the amount of time I would like with my family.

B13—I feel stimulated when I work with my colleagues.

B14—I deal very effectively with the problems of my patients and/or co-workers.

B16—In my work, I'm very relaxed when dealing with emotional problems.

B18—I feel exhilarated after working with or talking to patients.

Due to the small sample size, six conditional inference decision trees were created to measure responses of survey items^46–49^. Each decision tree includes the OC items (i.e., C9, C10, C19, C21, C22, and C30) as DVs and the burnout items (i.e., B1 through B18) as IVs. For example, the first decision tree includes question C30 (belief that the OC is positive) as the DV, while questions B1 (feeling emotionally drained after work), B5 (job interference with home-life), B6 (job interference with family time), B7 (irritation), B8 (anxiety), B9 (mood swings), B10 (feeling on-edge), B11 (fatigue), B12 (feeling at wits’ end), B15 (treating others as impersonal objects), and B17 (feeling callous toward others) were the IVs. The second decision tree also includes C30 as a DV but the remaining burnout questions, B2 (understanding patients’ feelings), B3 (understanding visitors’ feelings), B4 (taking one’s current job again), B13 (feeling stimulated when working with colleagues), B14 (effectively addressing problems), B16 (feeling relaxed when addressing emotional issues), and B18 (feeling exhilarated after working with or talking to patients), as IVs.

**Figure 14:**
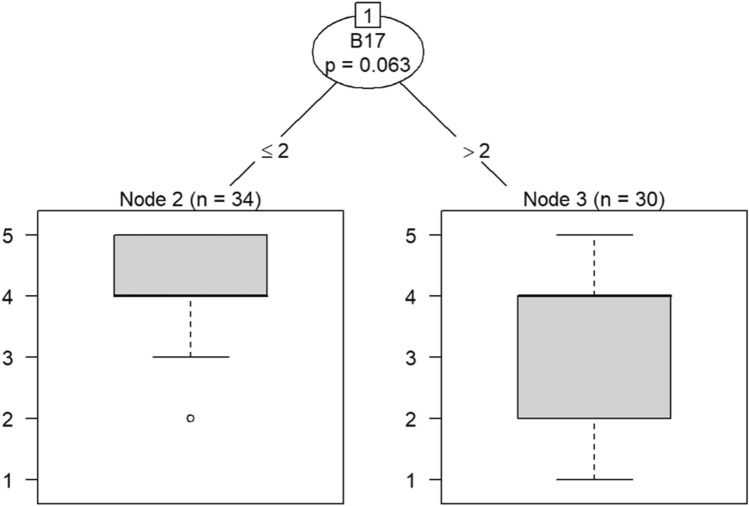
Conditional inference decision tree for Model 1—C30 (DV) and IVs B1, B5, B6, B7, B8, B9, B10, B11, B12, B15, and B17.

**Figure 15:**
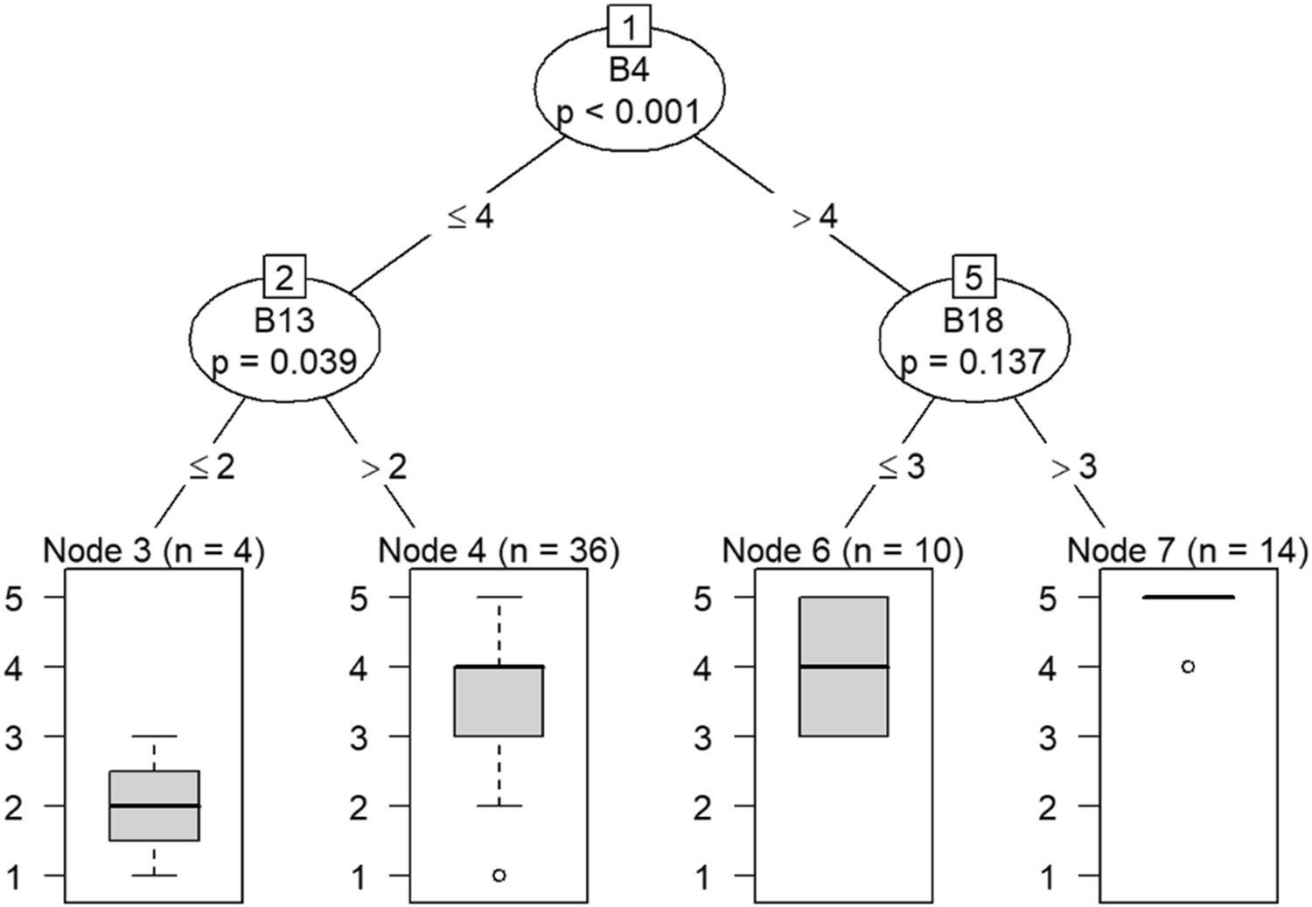
Conditional inference decision tree for Model 2—C30 (DV) and IVs B2, B3, B4, B13, B14, B16, and B18.

Model 1 in Fig. 14 shows that belief in a positive OC can predict callousness toward others. The decision tree in Fig. 15 shows that, when question C30 (belief that the OC was positive) was the DV, questions B4 (taking one’s current job again), B13 (feeling stimulated when working with colleagues), and B18 (feeling exhilarated after working with or talking to patients) were the only questions that could be predicted in a statistically significant way. Therefore, the model predicts that responses to C30 predicts responses to B4, B13, and B18. If the response to B4 is less than 4, meaning that participants were not likely to take their current job again, then their responses to B13 were likely to be either 3 or 4. Respondents would be either neutral or be somewhat likely to feel stimulated when working with colleagues. In a few instances, respondents were likely to not feel stimulated after working with colleagues if they were not likely to take their current job again.

**Figure 16:**
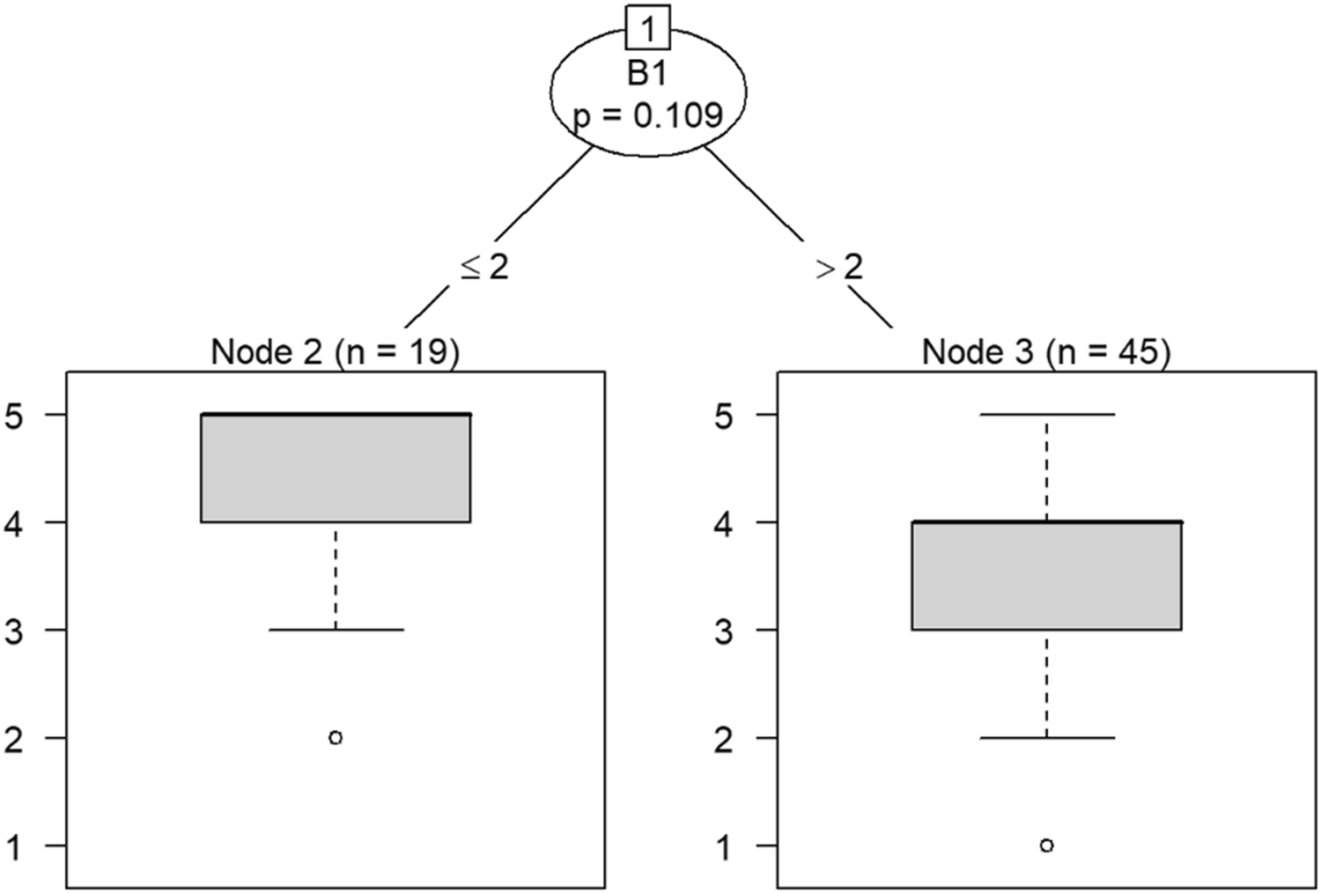
Conditional inference decision tree for Model 3—C9 (DV) and IVs B1, B5, B6, B7, B8, B9, B10, B11, B12, B15, and B17.

**Figure 17:**
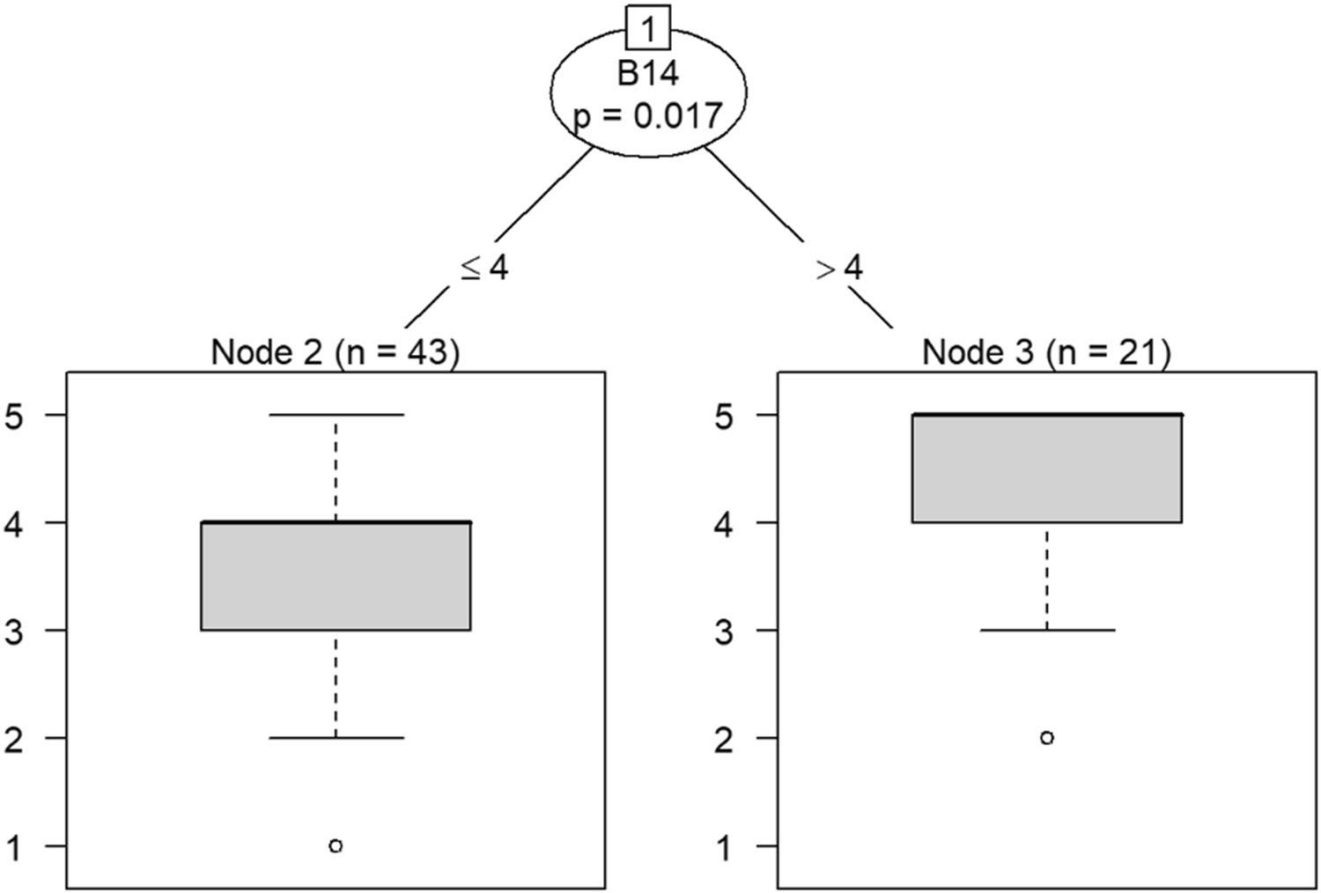
Conditional inference decision tree for Model 4—C9 (DV) and IVs B2, B3, B4, B13, B14, B16, and B18.

**Figure 18:**
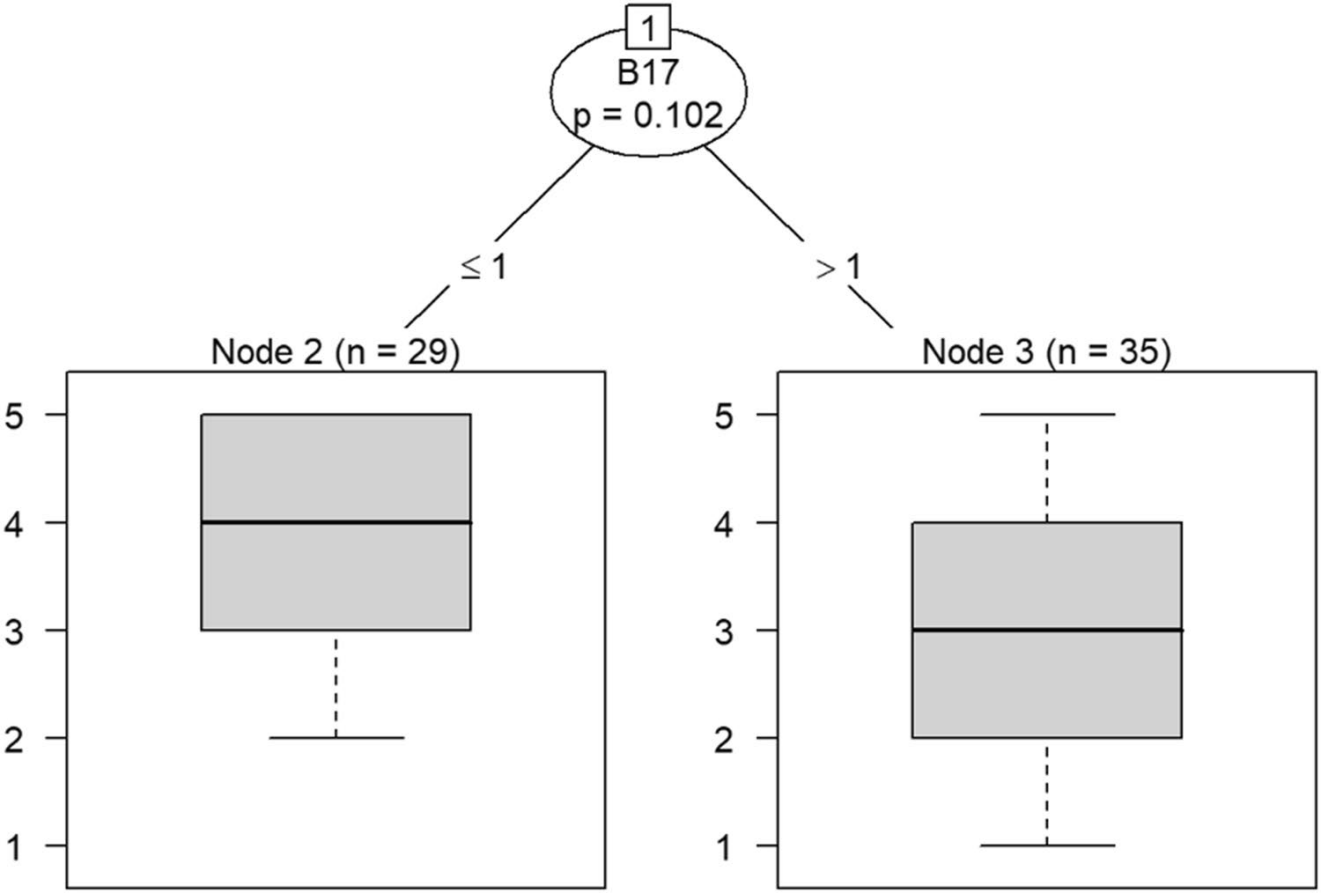
Conditional inference decision tree for Model 5—C10 (DV) and IVs B1, B5, B6, B7, B8, B9, B10, B11, B12, B15, and B17.

**Figure 19:**
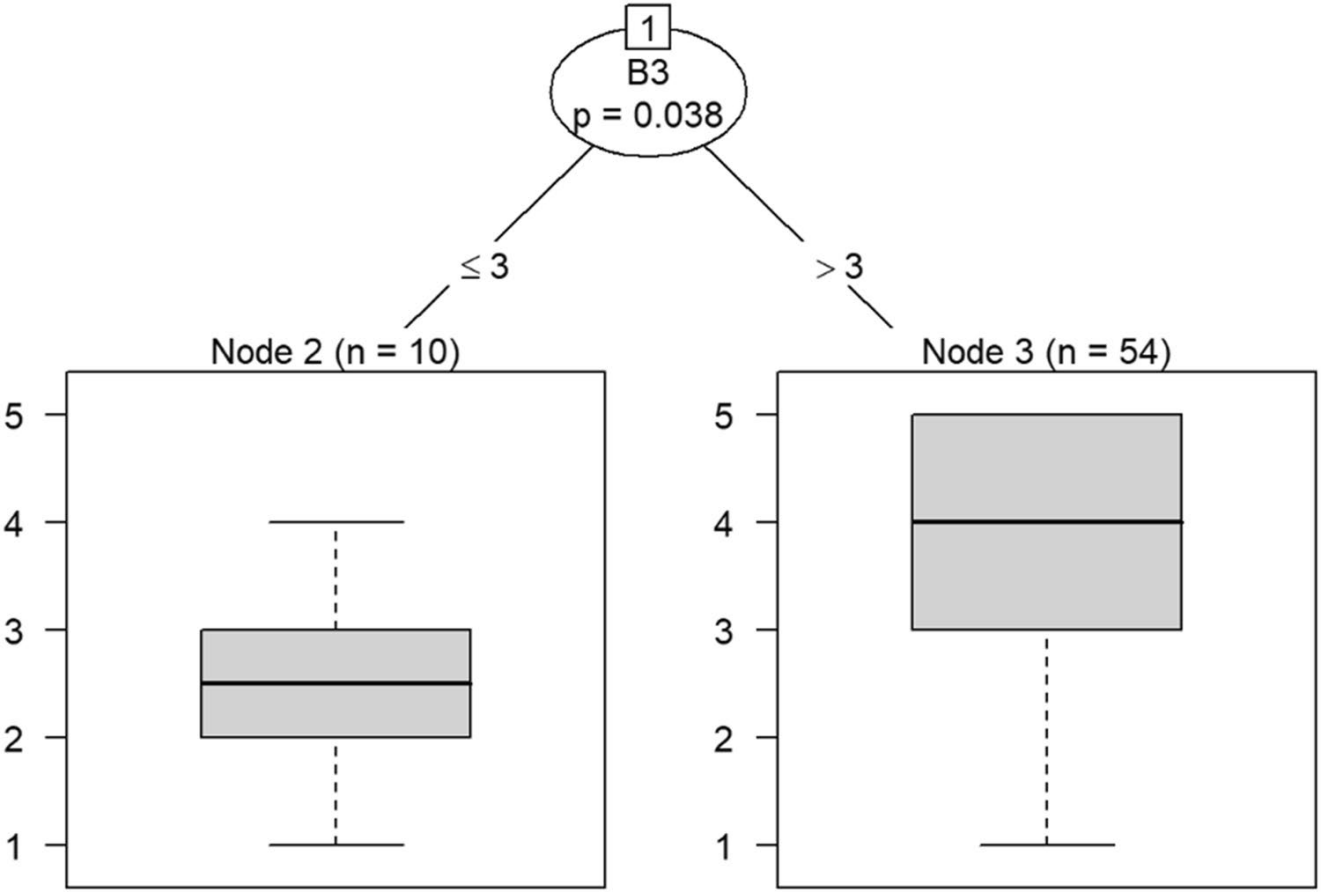
Conditional inference decision tree for Model 6—C10 (DV) and IVs B2, B3, B4, B13, B14, B16, and B18.

**Figure 20:**
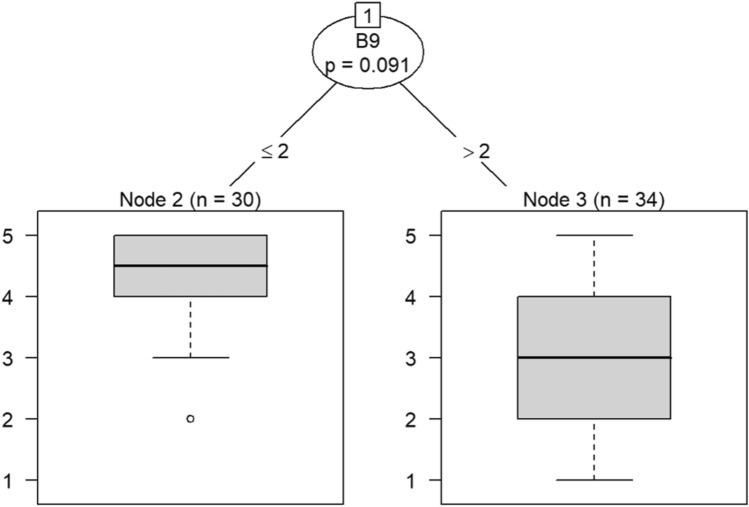
Conditional inference decision tree for Model 7—C19 (DV) and IVs B1, B5, B6, B7, B8, B9, B10, B11, B12, B15, and B17.

**Figure 21:**
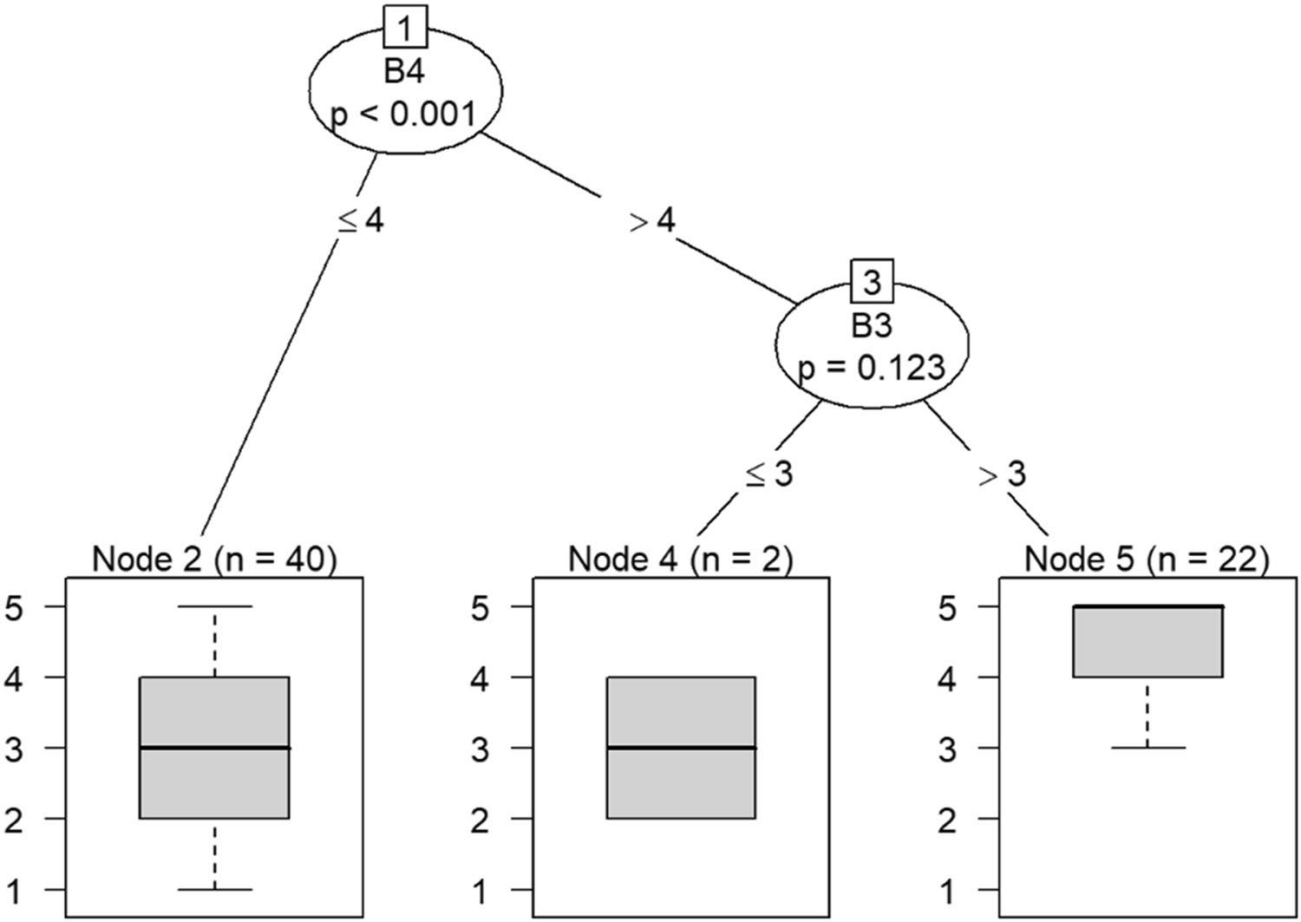
Conditional inference decision tree for Model 8—C19 (DV) and IVs B2, B3, B4, B13, B14, B16, and B18.

According to the decision tree models, question C9 (involving employees in decision-making) predicts responses to feeling emotionally exhausted after work and effectively addressing problems in Models 3 and 4 in Figs. 16 and 17, respectively. Question C10 (data-driven decision-making) predicts responses to understanding visitors’ feelings and becoming more callous toward others in Models 5 and 6 in Figs. 18 and 19, respectively. Question C19 (sacrificing patient safety to complete more work) predicts responses to understanding visitors’ feelings, retaking one’s job, and mood swings in Models 7 and 8 in Figs. 20 and 21, respectively. If a respondent was more likely to retake his or her job, then he or she would likely rate that he or she understood visitors’ feelings as a 4 or 5. However, two respondents were predicted to rate that they understood visitors’ feelings between 2 and 4.

**Figure 22:**
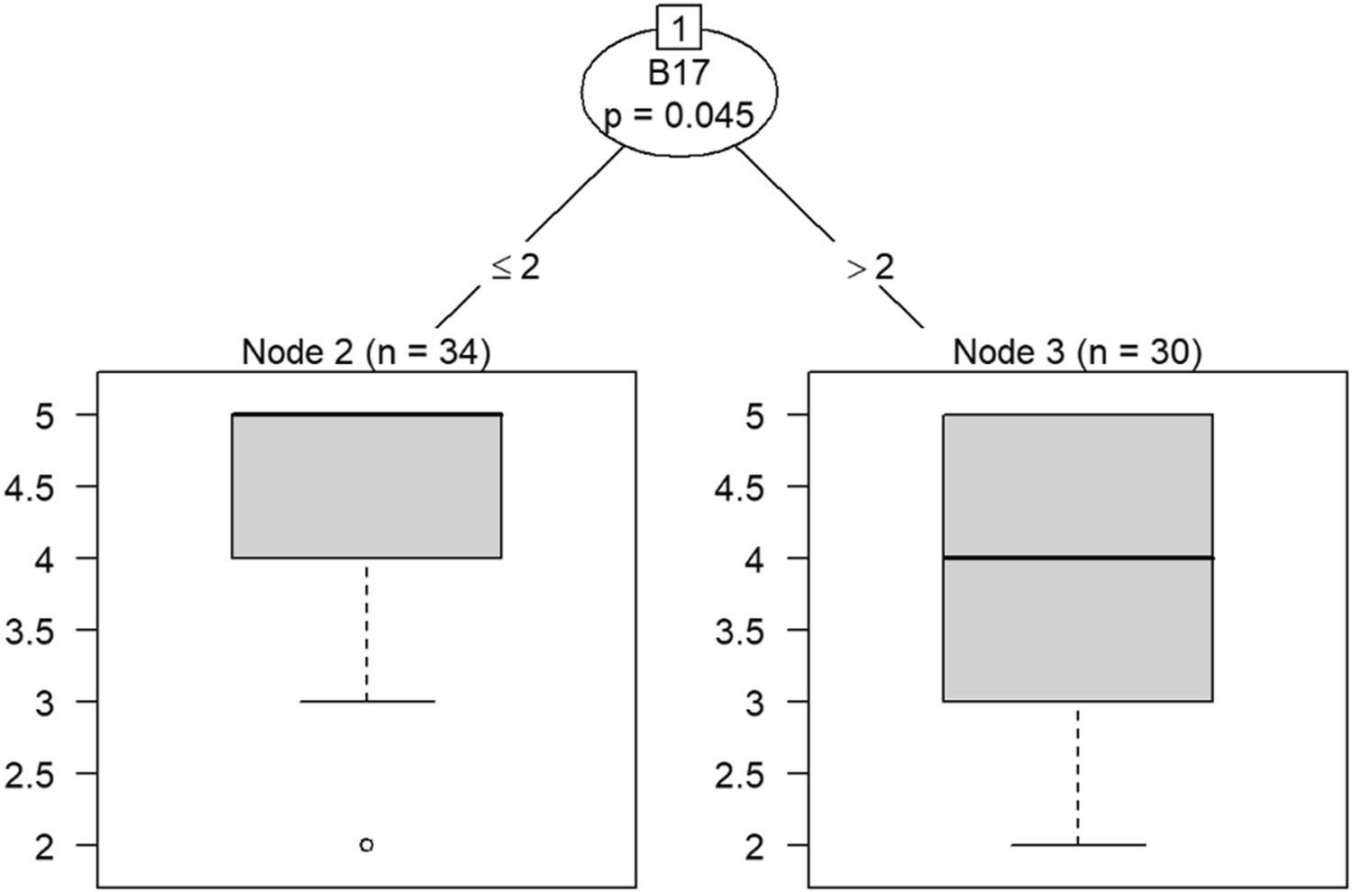
Conditional inference decision tree for Model 9—C21 (DV) and IVs B1, B5, B6, B7, B8, B9, B10, B11, B12, B15, and B17.

**Figure 23:**
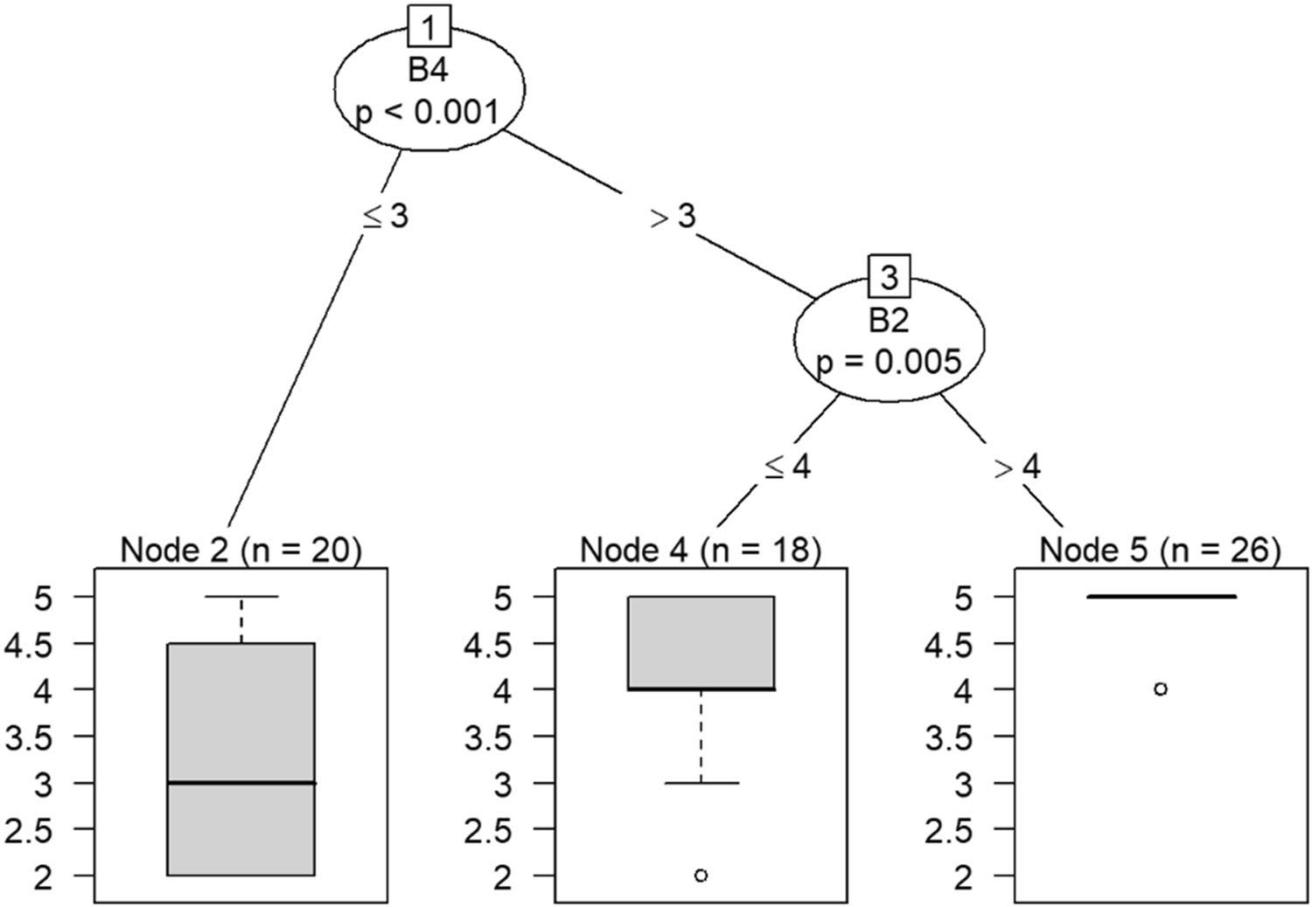
Conditional inference decision tree for Model 10—C21 (DV) and IVs B2, B3, B4, B13, B14, B16, and B18.

Question C21 (having the supplies and equipment needed to perform one’s job) predicts responses to feeling more callous toward others retaking one’s current job, and understanding patients’ feelings in Models 9 and 10 in Figs. 22 and 23, respectively. If a respondent rated their likelihood of retaking his or her job more than 3, then he or she would be more likely to understand patients’ feelings.

**Figure 24:**
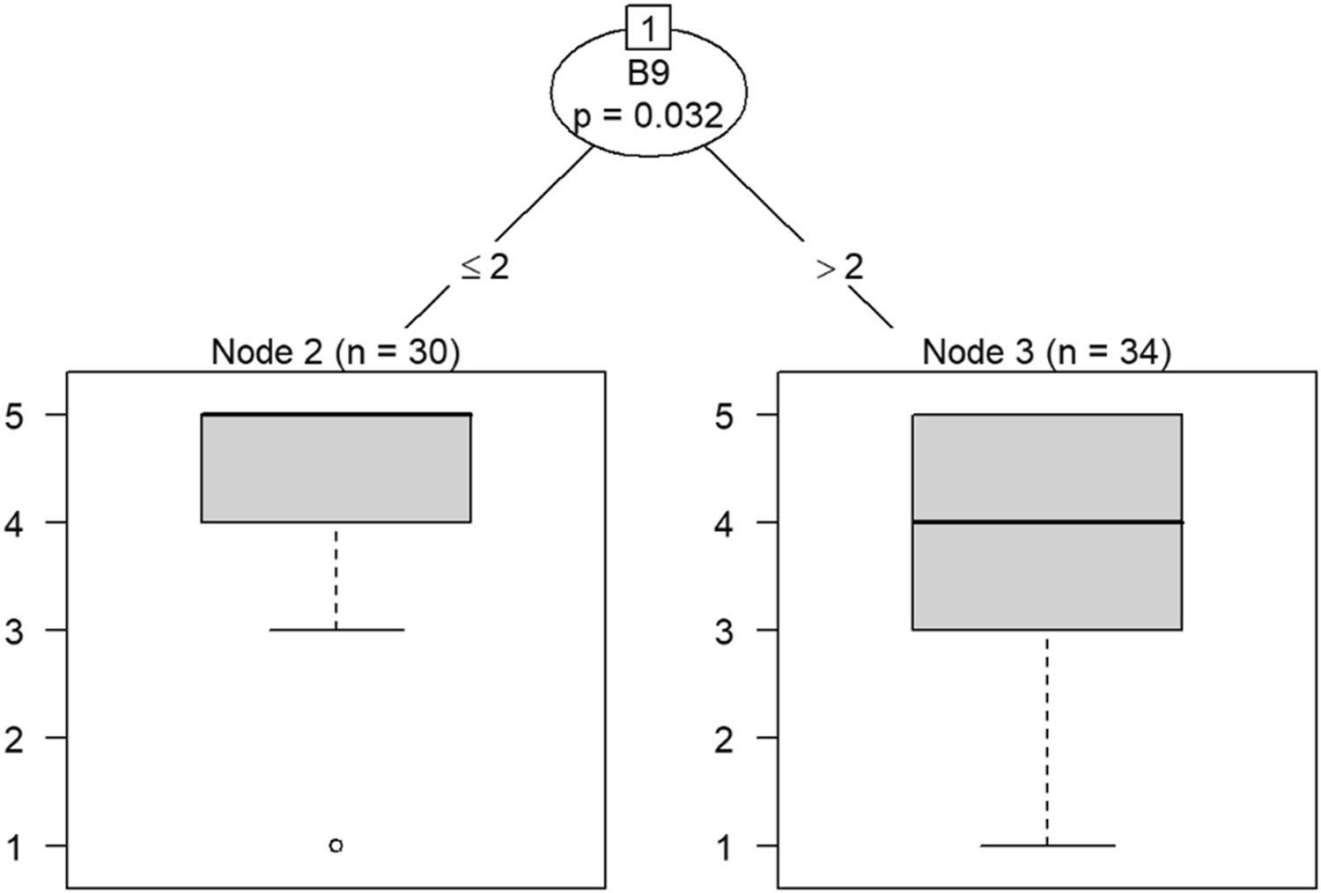
Conditional inference decision tree for Model 11—C22 (DV) and IVs B1, B5, B6, B7, B8, B9, B10, B11, B12, B15, and B17.

**Figure 25:**
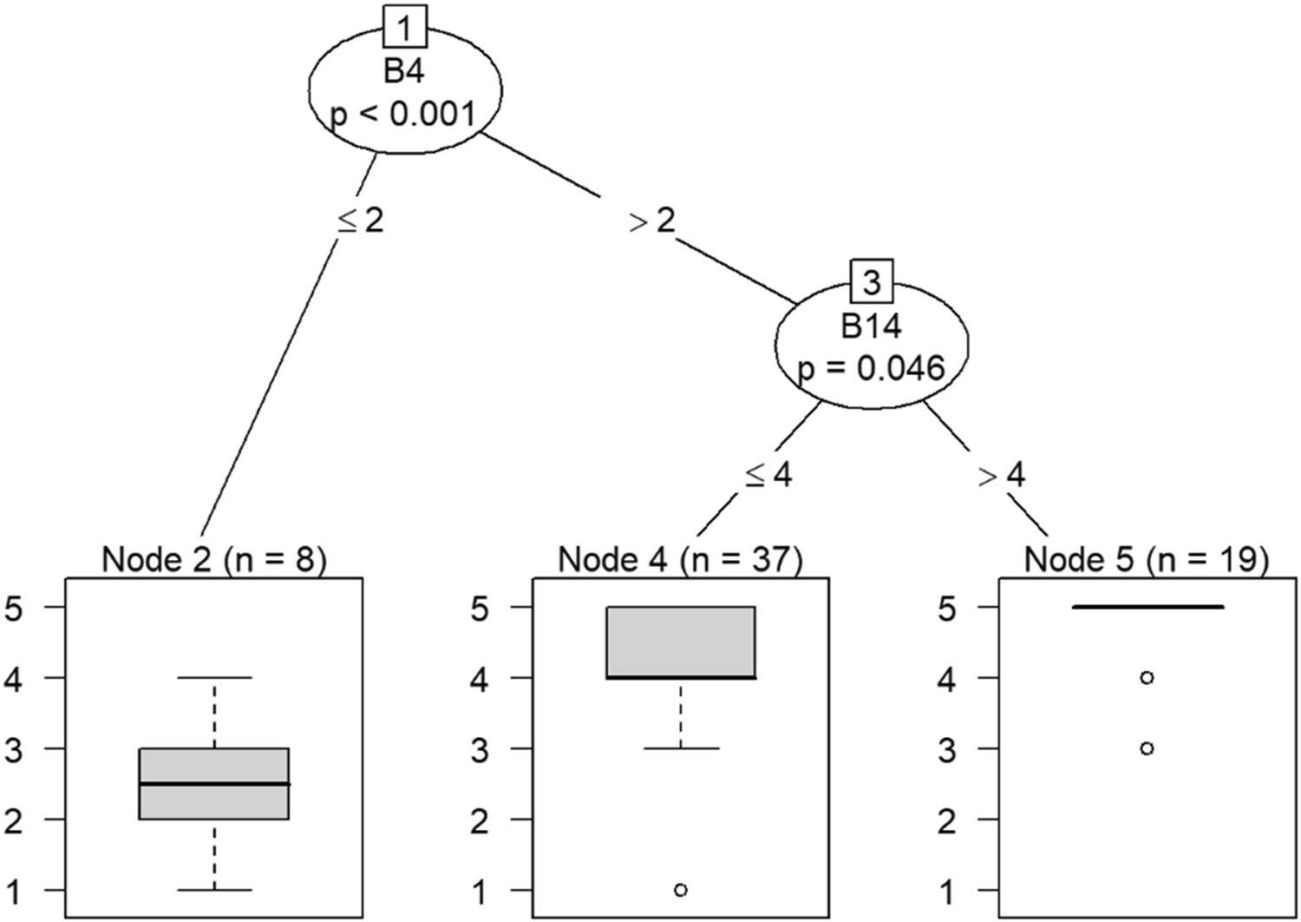
Conditional inference decision tree for Model 12—C22 (DV) and IVs B2, B3, B4, B13, B14, B16, and B18.

Question C22 (feeling comfortable reporting actual and potential safety issues) predicts responses to having mood swings, retaking one’s job, and effectively addressing problems in Models 11 and 12 in Figs. 24 and 25, respectively. If a respondent rated the likelihood of retaking his or her job as greater than 2, then he or she would be likely rate his or her ability to effectively address problems as 4 or 5.

If respondents were likely to take their current job again, then they were also likely to feel exhilarated after working with or talking to patients, as shown in nodes 6 and 7. Responses to question B4 also predict whether one has begun feeling more callous toward others since beginning his current job.

Tables 4, 5 and 6 show the performance of Models 1 through 12. Each model can potentially have a high error rate, with error rates of up to 1 Likert scale point. Additionally, low R-squared values denote low model fit.

**Table 4:** Models 1–4 performances.

	Model 1	Model 2	Model 3	Model 4
RMSE	1.04	1.16	1.25	1.37
R-squared	0.12	0.02	0.20	0.08

**Table 5:** Models 5–8 performances.

	Model 5	Model 6	Model 7	Model 8
RMSE	1.30	1.27	1.34	1.27
R-squared	0.12	0.04	0.07	0.14

**Table 6:** Models 9–12 performances.

	Model 9	Model 10	Model 11	Model 12
RMSE	1.15	1.15	1.38	1.46
R-squared	0.01	0.04	0.06	0.01

### Bayesian analysis

Due to the small sample size, a Bayesian analysis was also completed. Bayesian methods can be more robust with smaller sample sizes and allow for the incorporation of prior knowledge and provide credible intervals, offering a different perspective than traditional frequentist statistics.

A test of association produced a Bayes factor of 6:1 in favor of a relationship between employees perceiving the organizational culture as positive and the burnout symptoms of feeling emotionally drained after work, job interference with home life, job interference with family time, irritation, anxiety, mood swings, feeling on-edge, fatigue, feeling at one's wits' end, treating others as impersonal objects, and feeling callous toward others.

A second test of association produced a Bayes factor of 1:1 in favor of a relationship between employees perceiving the OC as positive and the engagement signs of understanding patients' feelings, understanding visitors' feelings, taking one's current job again, feeling stimulated when working with colleagues, effectively addressing problems, feeling relaxed when addressing emotional issues, and feeling exhilarated after working with or talking to patients.

Subsequent results of tests of association are in Tables 7 and 8. Notably, Matrix 4 shows a Bayes factor of 159:1 in favor of a relationship between employee involvement in decision-making and the aforementioned engagement signs. Additionally, Matrix 6 shows a Bayes factor of 56:1 in favor of a relationship between data-driven decision-making and employee engagement signs. Matrices 10 and 12 show Bayes factors of 0:1, which demonstrates a lack of a relationship between employees having all the necessary supplies to perform their jobs and engagement signs and employees feeling comfortable reporting potential safety issues and engagement signs, respectively.

**Table 7:** Matrices 1–8 Bayes factors.

	Matrix 1	Matrix 2	Matrix 3	Matrix 4	Matrix 5	Matrix 6	Matrix 7	Matrix 8
Bayes factor	6:1	1:1	3:1	159:1	2:1	56:1	3:1	9:1

**Table 8:** Matrices 9–12 Bayes factors.

	Matrix 9	Matrix 10	Matrix 11	Matrix 12
Bayes factor	4:1	0:1	7:1	0:1

## Discussion

The correlation matrices in Figs. 2 and 3 demonstrate a relationship between respondents believing that their OC is positive, such as employees valuing teamwork, collaboration, and information sharing, and several burnout factors. For example, the more likely patients believe that their OC is positive, the more likely they are to understand patients’ feelings, a result that corresponds with Silverman’s findings^50^. Respondents’ belief that OC is positive is statistically significant and negatively correlated with one’s job preventing one from spending time wanted with family, feeling fatigued when waking in the morning, and becoming more callous toward others since starting one’s current job. These results are corroborated by existing studies in different industries^51,52^. Results in the literature hold true for healthcare personnel.

Furthermore, the correlation matrices show statistically significant positive correlations between the belief that one’s OC is positive and several burnout factors, including understanding patients’ and visitors’ feelings, taking one’s current job again, feeling stimulated when working with colleagues, effectively addressing co-workers’ and patients’ issues, and feeling exhilarated after working with or talking to patients. These results correspond with the literature^53–56^. These results show that employees who perceive their OC to be positive are less likely to leave the organization, effectively address problems, and be engaged. The Bayesian analysis corroborates these results.

The correlation matrices and Bayesian analysis also show that having a supportive and data-driven OC in which employees are involved in evidence-based decision-making alleviates burnout symptoms, such as feeling emotionally drained after work, stimulated when working with others, and callous toward others. The results correspond with the literature that shows that burnout is positively correlated with decision-making difficulties^57,58^. However, this study adds to the literature by showing other ways in which decision-making is correlated with burnout.

Having an employee and patient safety-oriented culture is negatively correlated with several burnout symptoms, such as emotional exhaustion after work, mood swings, and irritability. This result aligns with studies that occurred in different settings, such as primary care offices^59–61^. Additionally, patient safety culture is positively correlated with work/life balance^59^. However, the previous studies focus on mainly patient safety and not employee safety. This study shows that focusing on employee safety is also negatively correlated to burnout symptoms. Therefore, leaders should focus on not only cultivating a culture of patient safety but also employee safety.

The decision tree in Models 1 and 2 and the Bayesian analysis show that belief that one’s OC is positive can predict employee engagement, turnover, and empathy. The decision trees’ predictions and the results of the Bayesian analysis align with the literature. Thus, leaders should ensure that employees believe that their OC is positive, such as by measuring perceptions of OC using surveys and strengthening the culture by supporting employees^62–64^. Doing so can protect against burnout^64–66^.

Furthermore, the decision trees featured in Models 3 to 12 provide insights into the capacity of a supportive, data-driven, safety-centric organizational culture (OC) to forecast burnout symptoms and associated outcomes. These outcomes encompass visitor and patient empathy, mood fluctuations, and turnover. The predictions rendered by the decision trees align with existing literature highlighting the correlations between OC and burnout. Nonetheless, this study extends this understanding by showcasing how OC can be instrumental in predicting specific burnout symptoms. Consequently, in scenarios where leaders identify signs of employee burnout, they are encouraged to evaluate the supportive, data-driven, and safety-oriented aspects of their OC. This approach enables leaders to employ objective OC measurements for the purpose of anticipating potential burnout among their employees.

Health systems’ leaders should ensure that they are creating a strong OC in which supervisors support their employees, employees feel adequately equipped to perform their jobs, employees are involved in decision-making, teamwork is prevalent, and co-workers treat one another well to mitigate burnout symptoms. Although several studies show a link between OC and burnout, none study all health systems’ employees with an emphasis on non-clinical, administrative employees. Therefore, this study’s results contribute to the literature by showing the relationship between OC and burnout among administrative and clinical health systems’ personnel.

### Study limitations

This study has several limitations despite its valuable contributions to both the academic literature and the industry. Firstly, the survey did not inquire about the name of the hospital to ensure participant anonymity, which hindered the ability to adjust results by hospital. Furthermore, due to the limited sample size, it was not feasible to aggregate results based on factors such as role, department, bed size, location, and hospital type. Each category within roles and departments garnered fewer than 30 responses, which is the minimum required for statistical significance.

A second limitation pertains to sample bias, primarily favoring administrative employees, despite efforts to mitigate bias by involving trade organizations in survey distribution. The survey was shared with over 100 trade organizations and health systems; however, only 67 responses were received due to several trade organizations and health systems either failing to respond to the first author (TJ) or declining participation for diverse reasons. Moreover, a total of 64 responses were used for analysis. The responses may not be representative of all health systems’ employees but mainly non-patient-facing employees. This limitation was mitigated by using conditional inference decision trees and conducting a Bayesian analysis, which is designed to test small samples, to corroborate the results of the decision tree models.

Further research endeavors should include an increased sample sizes to replicate and validate our discovered patterns. External validation is an essential step to appraise the broader applicability of findings across diverse populations. Future investigations employing more extensive and more diverse samples will contribute to the robustness and generalizability of the identified trends, fostering a deeper understanding of the phenomena under study.

## Conclusions

The research shows the importance of strengthening organizational culture to improve burnout through correlation and decision tree analysis. The models show that, when the OC is perceived as positive, employees are less likely to show burnout symptoms, such as feeling callous toward others and not understanding patients’ and visitors’ feelings. Therefore, health systems’ leaders should recognize that the burnout symptoms shown in the decision tree may be signs of an OC that should be improved.

### Contributions to the literature

This study contributes to literature in several ways. First, this is the first study to study mainly non-clinical and administrative health systems’ employees’ perceptions of OC and burnout. Previous studies about the perceptions of OC and burnout focus mainly on nurses and physicians with a non-clinical staff comprising a small subset of participants or no non-clinical participants. Second, this study is the first to use a decision tree model to show that perceptions of a positive OC can predict several burnout symptoms, such as callousness toward others and effective problem-solving skills.

### Contributions to the healthcare industry

The results of this study show leaders what burnout symptoms that OC can predict. For example, perceptions of a positive OC can predict whether employees empathize with visitors and patients. If patients do not empathize and are callous toward others, leaders should consider whether their OC is perceived as positive.

Second, this study shows how leaders can identify whether their employees are becoming burned out and how the OC can contribute to their burnout. For example, employees who treat patients and visitors with harsh language may be depersonalizing patients and visitors. Therefore, leaders can speak with employees privately and address the root cause of their depersonalization and possible burnout. Leaders may find that employees do not feel supported by their supervisors, which, as previous studies have shown, contributes to burnout. Leaders can then cultivate a culture where supervisors support their employees by providing them with the resources needed to work effectively and efficiently. They can also cultivate a culture in which work/life balance is emphasized; work/life balance is one aspect of OC that has been shown to impact burnout^67–69^. A lack of work/life balance shows that workload may be overwhelming for employees^70^. Leaders could model positive behaviors, such as using vacation time, and encourage employees to leave work at a certain time, especially for nonurgent requests.

## Data Availability

The datasets generated and/or analyzed during the current study are not publicly available due to participant anonymity and confidentiality, as specified in the IRB approval and informed consent, but are available from the corresponding author on reasonable request.
